# Subcutaneous Immunization with Inactivated Bacterial Components and Purified Protein of *Escherichia coli*, *Fusobacterium necrophorum* and *Trueperella pyogenes* Prevents Puerperal Metritis in Holstein Dairy Cows

**DOI:** 10.1371/journal.pone.0091734

**Published:** 2014-03-17

**Authors:** Vinícius Silva Machado, Marcela Luccas de Souza Bicalho, Enoch Brandão de Souza Meira Junior, Rodolfo Rossi, Bruno Leonardo Ribeiro, Svetlana Lima, Thiago Santos, Arieli Kussler, Carla Foditsch, Erika Korzune Ganda, Georgios Oikonomou, Soon Hon Cheong, Robert Owen Gilbert, Rodrigo Carvalho Bicalho

**Affiliations:** Department of Population Medicine & Diagnostic Sciences, College of Veterinary Medicine, Cornell University, Ithaca, New York, United States of America; Universidad Nacional de La Plata, Argentina

## Abstract

In this study we evaluate the efficacy of five vaccine formulations containing different combinations of proteins (FimH; leukotoxin, LKT; and pyolysin, PLO) and/or inactivated whole cells (*Escherichia coli*, *Fusobacterium necrophorum*, and *Trueperella pyogenes*) in preventing postpartum uterine diseases. Inactivated whole cells were produced using two genetically distinct strains of each bacterial species (*E. coli*, *F. necrophorum*, and *T. pyogenes*). FimH and PLO subunits were produced using recombinant protein expression, and LKT was recovered from culturing a wild *F. necrophorum* strain. Three subcutaneous vaccines were formulated: Vaccine 1 was composed of inactivated bacterial whole cells and proteins; Vaccine 2 was composed of proteins only; and Vaccine 3 was composed of inactivated bacterial whole cells only. Two intravaginal vaccines were formulated: Vaccine 4 was composed of inactivated bacterial whole cells and proteins; and Vaccine 5 was composed of PLO and LKT. To evaluate vaccine efficacy, a randomized clinical trial was conducted at a commercial dairy farm; 371 spring heifers were allocated randomly into one of six different treatments groups: control, Vaccine 1, Vaccine 2, Vaccine 3, Vaccine 4 and Vaccine 5. Late pregnant heifers assigned to one of the vaccine groups were each vaccinated twice: at 230 and 260 days of pregnancy. When vaccines were evaluated grouped as subcutaneous and intravaginal, the subcutaneous ones were found to significantly reduce the incidence of puerperal metritis. Additionally, subcutaneous vaccination significantly reduced rectal temperature at 6±1 days in milk. Reproduction was improved for cows that received subcutaneous vaccines. In general, vaccination induced a significant increase in serum IgG titers against all antigens, with subcutaneous vaccination again being more effective. In conclusion, subcutaneous vaccination with inactivated bacterial components and/or protein subunits of *E. coli*, *F. necrophorum* and *T. pyogenes* can prevent puerperal metritis during the first lactation of dairy cows, leading to improved reproduction.

## Introduction

Postpartum uterine diseases of dairy cows compromise animal welfare and may result in early removal from the herd or impaired reproductive performance. Puerperal metritis is defined by an abnormally enlarged uterus and a fetid, watery, red-brown uterine discharge associated with signs of systemic illness (decreased milk yield, dullness, or other signs of toxemia) and temperature >39.5°C within 21 d after parturition. Endometritis refers to inflammation of the uterus without systemic illness, happening later than 21 d postpartum [1]. In North America, metritis affects 10% to 20% of cows [2], whereas the incidence of endometritis is approximately 28%, ranging from 5.3% to 52.6% [3], [4]. Puerperal metritis is commonly treated with antibiotics like penicillin or third-generation cephalosporins. However, antibiotic resistance worldwide is recognized already as a top public health challenge facing the 21st century, and thus there is growing concern regarding the potential impact of extensive use of antibiotics in food animals, including later-generation cephalosporins [5], [6]. Overton and Fetrow (2008) reported the cost of each case of metritis to be approximately US$329–386, due to antibiotic treatment and the detrimental effects of metritis on reproductive performance, milk production, and survivability [7].

An efficacious vaccine against uterine diseases will have a significant positive impact on the dairy industry, limiting the use of antibiotics, and decreasing economic losses due to these disorders. Owing to the multifactorial nature of puerperal metritis and endometritis, a vaccine should likely be multivalent, including antigens from the most important etiological agents of uterine infections.

*Escherichia coli*, *Trueperella pyogenes* and *Fusobacterium necrophorum* are the primary bacterial causes of uterine diseases [8]–[10]. In the first days postpartum, *E. coli* is the predominant bacteria in the infected uterus, and is highly associated with uterine inflammation and impaired reproductive performance [11]–[13]. This early uterine contamination with *E. coli* leads to subsequent infection by *F. necrophorum* and *T. pyogenes* at 7 and >25 days postpartum, respectively [12], [14], which are associated with both metritis [9], [11], [12] and endometritis [15], [16].

Recently, two studies reported that FimH, an *E. coli* type 1 pilus adhesive protein that plays a critical role in adhesion to mannosides [17] and colonization of epithelial surfaces [18], is an important virulence factor that enables intrauterine *E. coli* to colonize the endometrium and initiate the uterine infection process [10], [19]. *E. coli* strains expressing type 1 pili containing FimH are the most important cause of urinary tract infection (**UTI**) in humans [20]. Immunization against FimH prevented *E. coli* colonization of the bladder mucosa in mice [21]. Additionally, the prevalence of liver abscesses, caused by *F. necrophorum* and *T. pyogenes* [22], was reduced successfully with a single dose of a vaccine containing inactivated *F. necrophorum* leukotoxin (**LKT**) and *T. pyogenes* pyolysin (**PLO**) [23]. Therefore, pre-partum immunization of cows with FimH, LKT and PLO may also reduce potentially the incidence of uterine diseases in dairy cattle.

Both intravaginal and systemic immunization against FimH, LKT, PLO, and relevant isolates of *E. coli*, *F. necrophorum* and *T. pyogenes* appear to be interesting strategies to successfully prevent bovine uterine diseases. Intravaginal immunization with a whole-cell vaccine has been shown to be very promising in the prevention of human urinary tract infection (UTI) [24], [25], increasing total vaginal and urinary IgG and IgA [24], and decreasing the risk of UTI in women [25]. On the other hand, results from other studies suggest that a systemic antibody response has a key role in local immunological protection in the bovine reproductive tract [26], [27], because most of the bovine intrauterine immunoglobulin is serum derived [26], and opsonic activity of cervicovaginal mucus from cows immunized systemically was higher than from cows immunized intravaginally [27].

Our hypothesis was that pre-partum immunization against relevant antigens for postpartum uterine diseases would prevent the occurrence of puerperal metritis and endometritis. For this purpose, we formulated 5 different vaccines (3 subcutaneous and 2 intravaginal) containing different combinations of proteins (FimH, LKT, PLO) and/or inactivated whole cells (*E. coli*, *F. necrophorum* and *T. pyogenes*). We report here that subcutaneous immunization effectively reduced the incidence of puerperal metritis, leading to enhanced reproductive performance.

## Materials and Methods

### Ethics statement

The field trial was conducted in a commercial dairy farm located near Ithaca, NY. This farm was selected because of its long working relationship with the Ambulatory and Production Medicine Clinic at Cornell University, and the trial was authorized by the farm owner, who was aware of all procedures. The research protocol was reviewed and approved by the Institutional Animal Care and Use Committee of Cornell University (Protocol number: 2011-0111).

### Inactivated bacterial components

*E. coli* strains 4612-2 and 12714-2 were selected because they possess virulence factors found to be associated with the occurrence of metritis (Bicalho et al., 2010). Each strain possess FimH and at least one of astA, cdt, kpsII, ibeA, and hly, which are virulence factors common to extraintestinal and enteroaggregative *E. coli*. Strains were grown aerobically on Luria-Bertani (LB) broth (Sigma-Aldrich) at 37°C. They were inoculated with 1% of an overnight culture and grown in 800 ml of medium, with agitation (150 rpm). For strain 12714-2, cells were harvested at 4 h, with an OD_600_ of 0.432 and 1.0×10^9^ CFU/ml; for strain 4612-2, cells were harvested at 3.5 h, OD_600_ of 0.473 and 1.2×10^9^ CFU/ml. The cultures were inactivated with 0.1% formalin for 12 h, and the cells were concentrated 4-fold (final volume of 200 ml), so 0.25 ml of each strain would be present in the final vaccine formulation, with approximately 10^9^ CFU per dose.

*Trueperella pyogenes* strains 10481-8 and 6375-1 were isolated from the uterine lumen of dairy cows. Strains were grown on VersaTREK REDOX 1 (Trek Diagnostic Systems, OH) in 7% CO_2_ at 37°C. Cells were harvested at 48 h, with 1.3×10^8^ and 0.5×10^8^ CFU/ml for strains 10481-8 and 6375-1, respectively. The cultures were inactivated with 0.1% formalin for 12 h, and 1 ml of each strain was added to the final vaccine formulation, with approximately 10^8^ CFU per dose.

*Fusobacterium necrophorum* strains 5663 and 513 were isolated from the uterine lumen of dairy cows. Strains were grown on VersaTREK REDOX 2 (Trek Diagnostic Systems, OH) anaerobically at 37°C. All cultures were inactivated with 0.1% formalin for 12 h before the cells were concentrated. Cells were harvested at 12 h, with 1.6×10^12^ and 1.8×10^12^ CFU/ml for strains 513 and 5663, respectively. The cultures were inactivated with 0.1% formalin for 12 h, and 0.01 ml of each strain was added to the final vaccine formulation, with approximately 10^10^ CFU per dose.

### Recombinant protein expression and purification

To generate the expression plasmids encoding PLO, The *PLO* gene, lacking the coding region for the predicted signal sequence, was amplified from *T. pyogenes* ATCC49698 genomic DNA by PCR with a 5′ primer containing an *Xho*I site (5′-ACAGCATCCTCGAGTGCCGGATTGGGAAAC-3′) and a 3′ primer containing an *Eco*RI site (5′-TGGAATTCCCTAGGATTTGACATTGT-3′) [28]. The 1.5-kb amplicon was digested with *Xho*I-*Eco*RI and cloned into *Xho*I-*Eco*RI-digested pTrcHisB (Invitrogen, NY).

The portion of the *FimH* gene encoding the signal peptide and the first 156 amino acids (the mannose-binding lectin domain, LD, [29]) of the mature protein was amplified from plasmid pET-22b(+)-F3-LD [30], provided by Dr. Evgeni Sokurenko, University of Washington, WA. The 5′ primer used contained a *Bam*HI site (5′-CGCGGATCCATGAAACGTGTTATTACCCTG-3′) and the 3′ primer contained a *Hin*dIII site (5′-CCCAAGCTTCTAGTGATGGTGATGGTGATGGCCGCCAGTAGGCACCAC-3′) and a six-histidine tag following the authentic sequence of the protein. The amplicon, approximately 0.6 kb, was digested with *Bam*HI-*Hin*dIII and cloned into *Bam*HI-*Hin*dIII-digested pTrcHisA (Invitrogen).

Bacteria were harvested after 5 hours of induction and cells were disrupted by two passages through a French pressure cell (Amicon) at 20,000 psi (138 Mpa), and the insoluble material was removed by centrifugation at 12,000×*g* for 30 min. His-tagged recombinant proteins were purified using TALON metal affinity resin (Clontech, CA) according to the manufacturer's instructions. Isolated pure protein fraction was concentrated using a fiber concentration/desalting system using a filter with a molecular weight exclusion of 10 kDa (Amicon ultra 100K, Millipore, MA) and subjected to SDS-PAGE (15%) using the Mini-PROTEAN Tetra Cell electrophoresis system (Bio-Rad, CA), following standard protocols. Protein concentration was determined by the Bradford method [31].

A total of 30 liters of culture was grown to produce a total of 321.24 mg of His-PLO. The final volume of His-PLO was 41 ml and the final concentration was 7.83 mg/ml. A total of 92 liters of culture was grown to produce 216.34 mg of FimH_1–156_-His. The final volume of FimH_1–156_-His was 172.5 ml and the concentration was 1.25 mg/ml.

#### Culture concentrated supernatant and affinity purification of Leukotoxin

*F. necrophorum* strain 6586 was grown in VersaTREK REDOX 2 for 12 h anaerobically at 37°C. The culture supernatant was concentrated at 4°C in a hollow fiber concentration/desalting system using a filter with a molecular weight exclusion of 100 kDa (Amicon ultra 100K, Millipore, MA). Affinity purification of LKT was performed to evaluate the concentration of LKT in the *F. necrophorum* 6586 culture concentrated supernatant, as described in [32]. Briefly, purified mAb F7B10 (3.5 mg) was coupled to 5 ml of Affi-Gel 10 affinity support (Bio-Rad, CA) and packed in a 1×20 cm column. The *F. necrophorum* 6586 culture concentrated supernatant was applied to the column, and non-binding materials were removed by passing 15 mL of 0.5 M NaCl in PBS through the column. Purified LKT was eluted with 0.2 M glycine-HCl (pH 3.0), immediately neutralized with NaOH, and washed and concentrated using an Amicon ultra 10K. Purity of the toxin was determined by SDS-PAGE.

A total of 10 L of *F. necrophorum* 6586 was grown to produce 220 mL of concentrated supernatant containing 0.186 mg/ml of LKT. The presence and concentration of LKT in the concentrated supernatant was determined by affinity purification.

### Vaccine formulation

Five different vaccine formulations were made: three subcutaneous vaccines (Vaccines 1–3) and two intravaginal vaccines (Vaccine 4–5). Vaccine 1 was composed of inactivated bacterial whole cells (*E. coli*, *T. pyogenes* and *F. necrophorum*) and proteins (FimH, PLO and LKT); Vaccine 2 was composed only of proteins (FimH, PLO and LKT); and Vaccine 3 was composed only of inactivated bacterial whole cells (*E. coli*, *T. pyogenes* and *F. necrophorum*). Vaccine 4 was composed of inactivated bacterial whole cells (*E. coli*, *T. pyogenes* and *F. necrophorum*) and proteins (FimH, PLO and LKT), and Vaccine 5 was composed only of proteins (PLO and LKT). The adjuvant for the subcutaneous vaccines was aluminum hydroxide (Rehydragel HPA, General Chemical, NJ). The adjuvant volume used in the subcutaneous vaccines was 25% of the final vaccine volume. Aluminum hydroxide was added to each component separately, and it was gently stirred overnight. The adjuvant for the intravaginal vaccines was 20 µg/dose of Cholera toxin (List Biological Laboratories, Inc., CA).

All vaccine components were tested for sterility before the final vaccine was assembled and bottled. Sterility was evaluated by culturing 100 µl of vaccine component aerobically in LB broth, aerobically in 7% CO_2_ on VersaTREK REDOX 1 and anaerobically on VersaTREK REDOX 2 at 37°C for 48 h. Components were considered contaminated if there was bacterial growth in any of the three culture media by the end of the incubation period.

Assessment of endotoxin levels was performed using the LAL Endpoint Assay (Hycult Biotech, The Netherlands) following the manufacturer's instructions. All vaccine formulations had endotoxin levels below 10^5^ EU/ml.

### Farm and management

Holstein pregnant heifers were enrolled from May 24, 2012 to August 16, 2012; the follow-up period continued until April 30, 2013. The farm milked 3,300 Holstein cows 3 times daily in a double 52-stall parallel milking parlor. All animals were subjected to the same immunization protocol prior and during the study period. At three months of age, all animals we immunized with Vista 5 SQ (Merck Animal Health, NJ), Covexin (Merck Animal Health, NJ), and Piliguard Pinkeye Triview (Merck Animal Health, NJ). They received a booster of each vaccine 2 weeks later. At 11 months, they received another dose of Vista 5 SQ. Furthermore, at 200 days of pregnancy, they were immunized with Triangle 9 (Boehringer Ingelheim Vetmedica, Inc., MO), and Covexin. At 250 and 264 they were immunized with J-Vac (Merial, GA), and Scourguard (Zoetis, NJ). Finally, at 35 DIM, they were immunized with Vista 5 SQ and J-Vac, and at the first pregnancy diagnosis date, they received another dose of J-Vac.

The heifers were housed in freestall barns with concrete stalls covered with mattresses and bedded with manure solids. All cows were offered a total mixed ration (TMR) consisting of approximately 55% forage (corn silage, haylage, and wheat straw) and 45% concentrate (corn meal, soybean meal, canola, cottonseed, and citrus pulp) on a dry matter basis of the diet. The diet was formulated to meet or exceed the NRC nutrient requirements for lactating Holstein cows weighing 650 kg and producing 45 kg of 3.5% fat corrected milk. The chemical composition of pre-fresh and fresh diets is presented in table S1. The reproductive management utilized a combination of Presynch [33], Ovsynch [34], Resynch [35], and detection of estrus, with 25% to 30% of cows bred via timed artificial insemination and the remainder bred after detection of estrus solely by activity monitors (ALPRO; DeLaval, Kansas City, MO).

### Treatment groups and Case definition

Prior to commencement of the study, statistical power and sample size calculations were performed. Based on the farm's average metritis incidence among primiparous cows, we assumed that the puerperal metritis incidence in the control group would be close to 30%. Considering a statistical power of 0.8, a *P*-value of 0.05, and that vaccination would decrease the puerperal metritis incidence to 10%, a sample size of 100 and 50 cows for control and treatment group, respectively, was considered sufficient.

Late pregnant heifers were enrolled on a weekly basis; inclusion criteria for enrollment were: 230±3 days of pregnancy, 629 to 734 days of age and body condition score (BCS) greater than 2.5. Heifers that were visually lame were not included in the study. A total randomized field trial study design was used; heifers were randomly allocated into one of six different treatment groups using the random number function of Excel (Microsoft, Redmond, MA). A total of 371 pregnant heifers were enrolled in the study; 105, 54, 53, 53, 53, and 53 heifers were randomly allocated to the control, Vaccine 1, Vaccine 2, Vaccine 3, Vaccine 4 and Vaccine 5 groups, respectively. Heifers assigned to the vaccine groups received two doses of vaccine: at 230±3 days of pregnancy and 260±3 days of pregnancy. Heifers assigned to the control group did not receive a placebo.

Information regarding ease of calving was gathered by farm workers, and a 5-point scale was used: EASE 1 was defined as calvings that occurred easily without assistance; EASE 2 was defined as unassisted, but more difficult than EASE 1, calvings; EASE 3 was defined as calvings requiring easy assistance from a person; EASE 4 was defined as vaginally delivered calvings requiring the calf position to be corrected or hard traction to be applied to deliver the calf; and EASE 5 was defined as calvings requiring fetotomy or caesarian section. Dystocia was defined as calving with EASE greater than 2.

Body condition scores were determined for all study cows at 230±3 days of gestation, 260±3 days of gestation, 2±1 days in milk (DIM), 6±1 DIM and at 35±3 DIM by a single investigator blinded to treatment group using a five-point scale with a quarter-point system as described by [36]. To obtain serum samples, blood was collected from a coccygeal vein/artery using a Vacutainer tube without anticoagulant and a 20 gauge×2.54 cm Vacutainer needle (Becton, Dickinson and Company, Franklin Lakes, NJ). All blood samples were transported to the laboratory on ice and spun in a centrifuge at 2,000×*g* for 15 min at 4°C; serum was harvested and frozen at −80°C. Serum samples were collected at 230±3 days of gestation, 260±3 days of gestation, 1±2 DIM, 6±1 DIM and 35±3 DIM. Rectal temperature was measured at 6±1 DIM using a digital thermometer (GLA M750, GLA Agriculture Electronics, CA) equipped with an angle probe (11.5 cm, 42°).

Cervical swabs were collected at 2±1 DIM and 6±1 DIM; cows were restrained and the perineum area was cleansed and disinfected with 70% ethanol solution. The swab was manipulated inside the cervix and exposed to uterine secretion. The swabs were kept inside a sterile vial at 4°C until processed in the laboratory. Swabs collected at 2±1 DIM were cultured aerobically on Chromagar (Difco) at 37°C and *E. coli* colonies were distinguished by a blue color; swabs collected at 6±1 DIM were cultured anaerobically on LKV agar (Anaerobe Systems) and *F. necrophorum* colonies were distinguished by morphology.

Retained placenta, puerperal metritis, ketosis, and clinical mastitis were diagnosed and treated by trained farm personnel who followed a specific diagnostic protocol designed by veterinarians from the Ambulatory and Production Medicine Clinic, Cornell University. Farm personnel were blinded to the treatments.

After parturition, cows were kept in the same pen until around 20 DIM. This pen was monitored by farm employees, and cows were submitted to a complete physical exam if they were showing signs of dullness and depression; cows with fetid, watery, red-brown uterine discharge accompanied with fever were diagnosed with puerperal metritis and treated by farm employees. Retained placenta was defined as a condition where cows failed to release their fetal membranes within 24 h of calving [37]. Puerperal metritis diagnosis by the research team was performed at 6±1 DIM. Puerperal metritis was defined as the presence of fetid, watery, red-brown uterine discharge and rectal temperature greater than 39.5°C [1]. Information regarding puerperal metritis diagnosis was not exchanged between farm personnel and the research team. Data regarding health traits and reproduction were extracted from the farm's DairyComp 305 database (Valley Agricultural Software, Tulare, CA).

Clinical endometritis diagnosis was evaluated at 35±3 DIM by visual inspection of a uterine lavage sample for the presence of purulent secretion as described [38]. To obtain uterine lavage samples, the cows were restrained, the perineum area was cleansed and disinfected with 70% ethanol, and a plastic infusion pipette was introduced into the cranial vagina and manipulated through the cervix into the uterus. A total of 20 ml of sterile saline solution was infused into the uterus and agitated gently, and a sample of the fluid was aspirated. The volume of recovered fluid ranged from 5 to 15 ml. All samples were visually scored by one investigator, who assessed the presence of a purulent or mucopurulent secretion in the uterine lavage sample. The score ranged from 0 to 2, with 0 indicating absence of a purulent or mucopurulent secretion, 1 indicating a bloody but not purulent sample, and 2 indicating the presence of pus in the lavage sample. Cows with a score of 2 were considered as diagnosed with clinical endometritis. Samples were kept on ice until they were cultured on Mueller–Hinton agar plates (BBL™) supplemented with 5% defibrinated sheep blood for 48 h aerobically in 5% CO_2_ at 38°C. Typical *T. pyogenes* colonies were distinguished by colony morphology, post-incubation hemolysis, and characteristic appearance on Gram's stain.

### Enzyme-linked immunosorbant assays (ELISAs)

Portions of the antigens produced for preparation of vaccines were used in ELISAs. *E. coli* strains were pooled together as a single antigen. The same was done for *F. necrophorum* and *T. pyogenes* strains.

The selected ELISA protocols were as follows. ELISA micro-titer plates (Greiner Bio-One, Germany) were coated with either 0.295 µg/ml of FimH_1–156_-His, 0.036 µg/ml of His-PLO, 0.186 µg/ml of LKT, 10^7^ cells/ml of *E. coli*, 10^10^ cells/ml of *F. necrophorum*, and 10^7^ cells/ml of *T. pyogenes* for anti-FimH, anti-LKT, anti-PLO, anti-*E. coli*, anti-*F. necrophorum*, and anti-*T. pyogenes* IgG assays, respectively. Serum samples were diluted in proportions of 1∶1000, 1∶5000, 1∶5000, 1∶150, 1∶500, and 1∶150 for anti-FimH, anti-LKT, anti-PLO, anti-*E. coli*, anti-*F. necrophorum*, and anti-*T. pyogenes* IgG assays, respectively. The optimal antigen and antibody concentrations were determined by performing the quantitative ELISA protocol with varying concentrations.

### Statistical analyses

Descriptive statistics analysis was undertaken in SAS using the FREQ procedure (SAS Institute INC., Cary, NC). To assess the effect of vaccination on the odds of RDPMET, FDPMET, endometritis, *E. coli*, *F. necrophorum*, and *T. pyogenes* culture outcomes, logistic regression models were fitted in SAS using the Logistic procedure. Contrasts were performed to compare the effect of subcutaneous vaccines composed by proteins (Vaccine 1 and Vaccine 2), and inactivated whole cells (Vaccine 1 and Vaccine 3) versus control. The effect of subcutaneous and intravaginal vaccines on reproduction was analyzed by Cox's proportional hazard using the proportional hazard regression procedure in SAS. To illustrate the effect of vaccination on reproduction, Kaplan-Meier survival analysis was performed using Medcalc version 10.4.0.0 (Mariakerke, Belgium). To assess the effect of vaccination on rectal temperature at 6±1 DIM, mixed general linear models were fitted to the data using JMP PRO9. To assess the effect of vaccination on ELISA detecting serum IgG against vaccine antigens, mixed general linear models were fitted to the data using JMP PRO9. For all models described above, independent variables and their respective interactions were kept when *P*<0.10 in an attempt to reduce the type II error risk while maintaining a stringent type I error risk of 5%. The variable *treatment* was forced into all statistical models even in the absence of statistical significance. Age in days at enrollment, BCS at enrollment, and dystocia were offered to all models.

## Results

### Descriptive statistics

Descriptive statistics regarding average age at enrollment (days), average BCS at enrollment and at 6±1 days postpartum, average gestation length at enrollment, and total number of animals enrolled are presented in Table 1. Only pregnant heifers were enrolled in this study, allowing us to have as little variation between animals as possible.

**Table 1:** Descriptive statistics of treatment groups.

	Control	Vaccine 1	Vaccine 2	Vaccine 3	Vaccine 4	Vaccine 5
Average age (days) at enrollment (± SE)	664 (3.72)	655 (5.2)	665 (5.24)	669 (5.24)	666 (5.24)	668 (5.24)
Average body condition score at enrollment (± SE)	3.71 (0.03)	3.76 (0.05)	3.74 (0.05)	3.65 (0.05)	3.72 (0.05)	3.66 (0.05)
Average body condition score at 6±1 (± SE)	3.5 (0.02)	3.49 (0.03)	3.52 (0.03)	3.49 (0.03)	3.44 (0.03)	3.50 (0.03)
Average days of gestation at enrollment (± SE)	230 (0.21)	230 (0.29)	230 (0.29)	230 (0.29)	230 (0.29)	230 (0.29)
Total enrolled animals (%)	105 (28.3)	54 (14.5)	53 (14.3)	53 (14.3)	53 (14.3)	53 (14.3)

### Effect of vaccination on incidence of researcher diagnosed puerperal metritis (RDPMET), farm diagnosed puerperal metritis (FDPMET), and rectal temperature at 6±1 DIM

The effect of vaccination on the incidence of RDPMET is presented in Table 2. When evaluated separately, there was no difference between incidence of RDPMET between treatment groups (*P*-value = 0.153). However, when vaccines were evaluated grouped as either subcutaneous or intravaginal vaccines, the subcutaneous vaccines were associated with a significant reduction in the incidence of RDPMET (*P*-value = 0.018). Additionally, contrasts showed a significant reduction on the incidence of RDPMET for cows subcutaneously immunized with inactivated whole cells (Vaccine 1 & 3, *P*-value = 0.035).

**Table 2:** Effects of different vaccine formulations on incidence of researcher diagnosed puerperal metritis.

Model and variables	Puerperal metritis incidence (%)	Coefficients (SE)	Odds ratio (95% CI)	Individual *P*-value	Overall *P*-value
**Model 1**					
Control	12.1	Ref.	baseline		
Vaccine 1	6.2	−0.14 (0.56)	0.44 (0.11–1.67)	0.226	
Vaccine 2	4.1	−0.73 (0.65)	0.24 (0.05–1.17)	0.078	
Vaccine 3	2.0	−1.32 (0.87)	0.13 (0.02–1.08)	0.060	0.153
Vaccine 4	13.5	0.68 (0.43)	0.99 (0.35–2.78)	0.989	
Vaccine 5	14.0	0.80 (0.43)	1.12 (0.40–3.12)	0.832	
Intercept		−2.15 (0.27)			
**Model 2**					
Control	12.1	Ref.	baseline		
Subcutaneous	4.1	−0.90 (0.32)	0.27 (0.09–0.75)	0.013	0.018
Intravaginal	13.7	0.47 (0.26)	1.05 (0.45–2.46)	0.905	
Intercept		−1.88 (0.22)			
**Contrasts**					
Control	12.1	Ref.	baseline		
Vaccine 1 & 2	5.1	−1.12 (0.57)	0.32 (0.10–1.01)	0.051	
Vaccine 1 & 3	4.1	−1.42 (0.67)	0.24 (0.06–0.91)	0.035	

Vaccines were evaluated separately in Model 1, and grouped in Model 2. Age in days, dystocia, and body condition score at enrollment were offered to both models.

The effect of vaccination on incidence of FDPMET is present in Table 3. When the vaccines were evaluated separately, the incidence of FDPMET tended to be different among the treatments (*P*-value = 0.056). When compared to control, Vaccine 1 reduced the incidence of FDPMET (*P*-value = 0.019). Additionally, when the vaccines were evaluated grouped as subcutaneous or intravaginal vaccines, the subcutaneous vaccines were associated with a significantly lower odds of FDPMET (*P*-value = 0.034). Furthermore, contrasts showed a significant reduction on the incidence of RDPMET for cows subcutaneously immunized with proteins (Vaccine 1 & 2, *P*-value = 0.010), and inactivated whole cells (Vaccine 1 & 3, *P*-value = 0.026).

**Table 3:** Effects of different vaccine formulations on incidence of farm diagnosed puerperal metritis.

Model and variables	Puerperal metritis incidence (%)	Coefficients (SE)	Odds ratio (95% CI)	Individual *P*-value	Overall *P*-value
**Model 1**					
Control	27.6	Ref.	baseline		
Vaccine 1	11.1	−0.73 (0.38)	0.31 (0.12–0.82)	0.019	
Vaccine 2	17.0	−0.29 (0.33)	0.49 (0.21–1.14)	0.100	
Vaccine 3	20.7	−0.01 (0.31)-	0.65 (0.29–1.45)	0.297	0.056
Vaccine 4	34.0	0.67 (0.28)	1.27 (0.62–2.62)	0.504	
Vaccine 5	19.2	−0.08 (0.32)	0.60 (0.27–1.37)	0.226	
Intercept		−1.06 (0.18)			
**Model 2**					
Control	27.6	Ref.	baseline		
Subcutaneous	16.2	−0.46 (0.18)	0.48 (0.26–0.88)	0.018	0.034
Intravaginal	26.7	0.18 (0.18)	0.91 (0.49–1.68)	0.766	
Intercept		−0.91 (0.17)			
**Contrasts**					
Control	27.6	Ref.	baseline		
Vaccine 1 & 2	14.0	−0.93 (0.36)	0.39 (0.19–0.80)	0.010	
Vaccine 1 & 3	15.9	−0.35 (0.35)	0.45 (0.22–0.91)	0.026	

Vaccines were evaluated separately in Model 1, and grouped in Model 2. Age in days, dystocia, and body condition score at enrollment were offered to both models.

**Figure 1:**
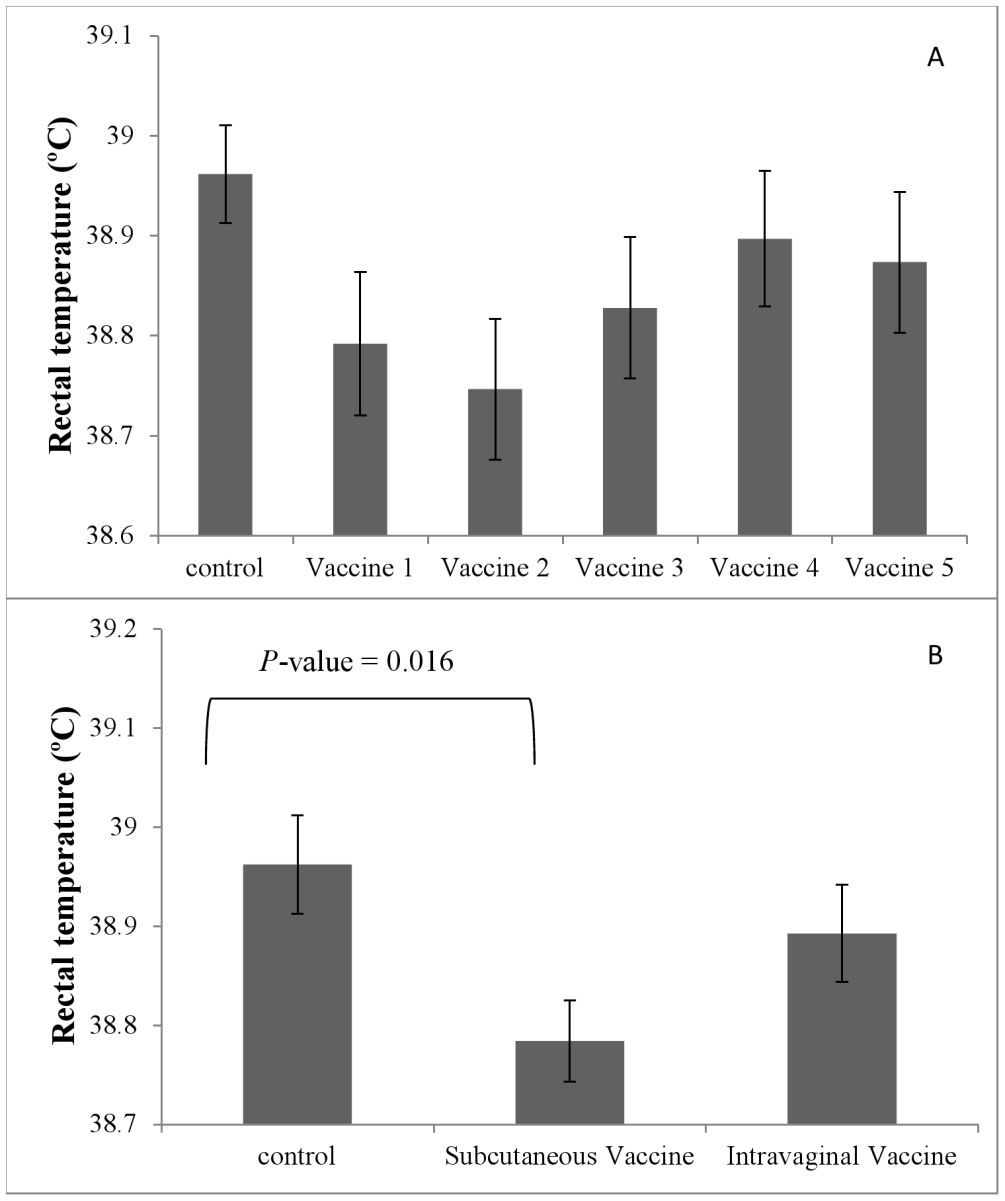
Effect of vaccination on rectal temperature at 6±1 DIM. Vaccines were evaluated separately (A, *P*-value = 0.14), and grouped (B, *P*-value = 0.018). Standard errors of the means are represented by the error bars.

The effect of vaccination on rectal temperature at 6±1 DIM is presented in Figure 1. Rectal temperature was not statistically different among the treatment groups when the vaccines were evaluated separately (*P*-value = 0.14); rectal temperature was 38.96°C (SEM = 0.05), 38.79°C (SEM = 0.07), 38.75°C (SEM = 0.07), 38.83°C (SEM = 0.07), 38.90°C (SEM = 0.07), and 38.87°C (SEM = 0.07) for control, Vaccine 1, Vaccine 2, Vaccine 3, Vaccine 4, and Vaccine 5 cows, respectively. However, rectal temperature was statistically different between the treatment groups when the vaccines were evaluated grouped as control, subcutaneous vaccines or intravaginal vaccines (*P*-value = 0.018); rectal temperature was 38.96°C (SEM = 0.05), 38.78°C (SEM = 0.04), and 38.89°C (SEM = 0.05) for control, subcutaneous vaccinated, and intravaginally vaccinated cows, respectively. Subcutaneous vaccination was associated with a significant reduction in rectal temperature at 6±1 DIM.

### Effect of vaccination on incidence of endometritis and uterine secretion culture outcomes

Vaccines were not effective in preventing endometritis, when evaluated separately or when grouped as subcutaneous and intravaginal vaccines (*P*-value = 0.99). Endometritis incidence was 8.6%, 7.9%, 12.1%, 7.5%, 9.1%, and 9.8% for control, vaccine 1, vaccine 2, vaccine 3, vaccine 4, and vaccine 5, respectively. The incidence of endometritis was 9.0% and 9.5% for subcutaneous and intravaginal vaccines, respectively. Additionally, there was no significant effect of vaccination on the likelihood of intrauterine bacterial contamination (Table 4).

**Table 4:** Effects of different vaccine formulations on incidence of intrauterine *Escherichia coli* at 2±1 DIM, *Fusobacterium necrophorum* at 6±1 DIM and *Trueperella pyogenes* at 35±3 DIM.

Model and variables	Cows positive for intrauterine culture (%)	Coefficients (SE)	Odds ratio (95% CI)	*P*-value
**Model 1**	***E. coli***			
Control	55.0	Ref.	baseline	
Vaccine 1	47.1	−0.01 (0.26)	0.73 (0.37–1.45)	
Vaccine 2	46.1	−0.09 (0.25)	0.67 (0.34–1.34)	
Vaccine 3	40.4	−0.36 (0.26)	0.52 (0.26–1.03)	0.57
Vaccine 4	50.9	0.10 (0.25)	0.82 (0.42–1.61)	
Vaccine 5	50.0	0.05 (0.26)	0.78 (0.39–1.55)	
Intercept		1.91 (1.14)		
**Model 2**	***E. coli***			
Control	55.0	Ref.	baseline	
Subcutaneous	44.5	−0.23 (0.14)	0.63 (0.38–1.06)	0.21
Intravaginal	50.5	−0.01 (0.46)	0.80 (0.46–1.40)	
Intercept		1.89 (1.13)		
**Model 3**	***F. necrophorum***			
Control	49.0	Ref.	baseline	
Vaccine 1	36.0	−0.39 (0.26)	0.59 (0.29–1.18)	
Vaccine 2	48.0	0.11 (0.26)	0.96 (0.49–1.90)	
Vaccine 3	48.0	0.11 (0.26)	0.96 (0.49–1.90)	0.76
Vaccine 4	47.2	0.07 (0.25)	0.93 (0.48–1.81)	
Vaccine 5	44.0	−0.05 (0.26)	0.82 (0.41–1.62)	
Intercept		−0.19 (0.11)		
**Model 4**	***F. necrophorum***			
Control	49.0	Ref.	baseline	
Subcutaneous	44.0	−0.09 (0.14)	0.82 (0.49–1.36)	0.74
Intravaginal	45.6	−0.02 (0.16)	0.87 (0.50–1.52)	
Intercept		−0.15 (0.11)		
**Model 5**	***T. pyogenes***			
Control	14.5	Ref.	baseline	
Vaccine 1	5.3	−0.80 (0.63)	0.30 (0.06–1.46)	
Vaccine 2	21.2	0.77 (0.42)	1.44 (0.48–4.32)	
Vaccine 3	12.5	0.05 (0.46)	0.70 (0.21–2.30)	0.37
Vaccine 4	12.1	0.06 (0.50)	0.70 (0.20–2.52)	
Vaccine 5	7.3	−0.49 (0.54)	0.41 (0.10–1.61)	
Intercept		−16.55 (5.48)		
**Model 6**	***T. pyogenes***			
Control	14.5	Ref.	baseline	
Subcutaneous	12.6	0.01 (0.26)	0.74 (0.30–1.82)	0.50
Intravaginal	9.5	−0.32 (0.31)	0.53 (0.19–1.53)	
Intercept		−16.66 (5.48)		

Vaccines were evaluated separately in Model 1, Model 3 and Model 5; and grouped in Model 2, Model 4 and Model 6. Age in days, dystocia, and body condition score at enrollment were offered to both models.

### Effect of vaccination on reproduction

**Figure 2:**
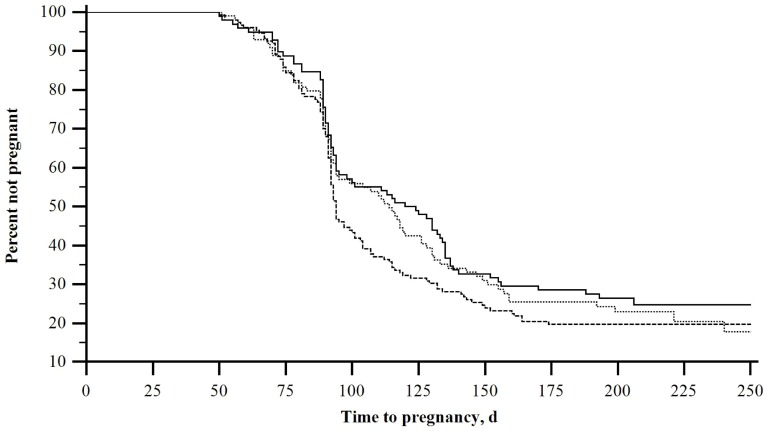
Effect of subcutaneous and intravaginal vaccines on reproduction. The median calving-to-conception interval for subcutaneously vaccinated cows (inner interrupted line), intravaginally vaccinated cows (middle interrupted line), and control cows (solid line) was 94, 114, and 120 respectively. (*P-*value = 0.04).

Cows that received subcutaneous vaccination were 1.36 times more likely to conceive when compared to control cows (*P*-value = 0.04, Figure 2). However, for cows that received intravaginal vaccines, the likelihood of conceiving was not statistically different from control cows (Hazard ratio = 1.12, *P*-value = 0.46). Age in days at enrollment and BCS at enrollment were retained in the model for this analysis (*P*-value = 0.02 and 0.01, respectively).

### Serological responses to vaccination

**Figure 3:**
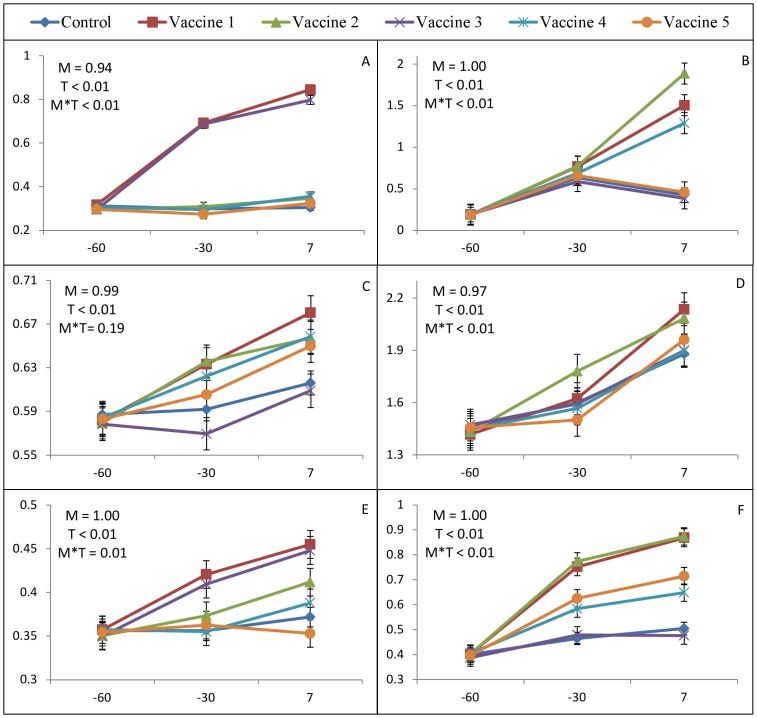
Effect of vaccination on ELISA-detected serum IgG against *E. coli* (A), FimH (B), *F. necrophorum* (C), LKT (D), *T. pyogenes* (E), and PLO (F). X-axis represents days relative to calving, while Y-axis represents OD_650_ of ELISA-detected serum IgG against several antigens. Standard errors of the means are represented by the error bars.

The effect of vaccination on ELISA-detected serum IgG against several antigens is presented in Figure 3. Vaccine 1 and 2 increased serum IgG titers against *E. coli*, while cows from all other treatment groups did not respond to this antigen. Additionally, cows vaccinated with vaccines 1, 2, and 4 had increased IgG levels against to FimH. However, it seems that the animals naturally responded to LKT and *F. necrophorum*, because all animals have elevated IgG titers against these antigens after parturition. Cows vaccinated with vaccine 1 and 3 had increased IgG levels against *T. pyogenes*, while vaccine 1 and 2 had increased IgG titers against PLO.

## Discussion

We evaluated here the effects of 5 different vaccine formulations (3 subcutaneous vaccines and 2 intravaginal vaccines) containing different combinations of proteins (FimH, LKT, PLO) and inactivated whole cells (*E. coli*, *F. necrophorum* and *T. pyogenes*) on the uterine health of dairy cows. We demonstrated that subcutaneous vaccination significantly decreased the incidence of puerperal metritis, whereas intravaginal vaccination was not effective.

Puerperal metritis is characterized by inflammation of the entire thickness of the uterine walls, and is associated with signs of systemic illness such as dullness, decreased milk yield and fever [1]. The signs of puerperal metritis (presence of fetid, watery, red-brown uterine discharge and rectal temperature greater than 39.5°C) used for the diagnosis of metritis in this study is widely used by researchers and veterinarians. In a recent study, it was reported that there is a considerable inconsistency between observers to classify animals as healthy or metritic based on the assessment of vaginal discharge odor [39], suggesting that the classification of disease based on the signs used is prone to errors. However, we expect that errors occurred equally among all treatment groups. When diagnosed by our research group, puerperal metritis incidence was 12.1% and when diagnosed by farm workers it was 27.6%. This discrepancy can be attributed to the period during which the cows were monitored; whereas farm workers monitored the cows daily during their first 20 days after parturition, the research team examined the cows only at 6±1 days after calving. Cows were examined at this time point because metritis peaks in the first 7 days after calving [40]. However, it is important to highlight that, in general, the effect of vaccination on puerperal metritis was consistent between the research group's and the farm workers' diagnoses; subcutaneous vaccination significantly lowered the incidence of puerperal metritis, whereas intravaginal vaccine was not effective in preventing the disease.

*E. coli* and *F. necrophorum* are gram-negative bacteria, characterized by the presence of lipopolysaccharide (**LPS**) in their outer membrane, and are known etiological agents of puerperal metritis; LPS is known to cause increased body temperature in cattle [41]. Although vaccination did not significantly decrease the percentage of cows that were positive for intrauterine *E. coli* and *F. necrophorum*, subcutaneously vaccinated cows did have a lower rectal temperature at 6±1 DIM. The differences of the rectal temperature between treatment groups was small; however, control cows had higher rectal temperature, suggesting that more cows in the control group were found with fever. This suggests that, even in the presence of bacteria in the uterus, immunized cows were less likely to develop systemic signs caused by LPS released from *E. coli* and *F. necrophorum*. It is known that reducing the bacterial load of *E. coli* decreases the severity of the disease [1]; therefore, we can also speculate that immunization decreased the pathogen-load inside the uterus. However, further investigation is needed to address questions regarding the mechanisms of action of the vaccine.

The relationship between poor immune status around calving and uterine diseases is already well established [42]–[46]. Recruitment of polymorphonuclear cells (**PMNs**) to the endometrial surface and the uterine lumen is critical for the immune defense of the uterus [13]. A vaccine against uterine diseases would have great potential for enhancing the immune status around parturition, by inducing production of pathogen-specific immunoglobulins in bovine endometrial secretions, which would act by lysing bacteria, by serving as opsonins to enhance phagocytosis, and by stimulating the complement pathways [47].

Although it was found that subcutaneous immunization effectively prevented puerperal metritis, we did not observe the same effect on endometritis. Metritis and endometritis appear to be linked uterine diseases; however, metritis is not necessary for the development of endometritis [13], [48]. This finding suggests that immunization against the targeted, while important to prevent puerperal metritis, was not effective to decrease the incidence of endometritis. Further investigation is needed to evaluate if addition of others antigens to these vaccines would contribute to prevention of endometritis. A potential candidate would be the fimbriae subunit FimA; the gene FimA is highly prevalent in *T. pyogenes* isolated from the uterus of dairy cows [49], and it was associated with development of metritis [12], [49] and endometritis [12].

Mucosal immune responses can be effectively induced by the administration of vaccines onto mucosal surfaces, whereas subcutaneous and intramuscular vaccines typically fail to induce mucosal immunity, and are less effective in preventing infection of mucosal surfaces [50]. Promising results regarding prevention of human UTI by intravaginal immunization with a whole-cell vaccine have already been reported [24], [25]. However, it is not known how local synthesis of specific antibodies by uterine antibody-secreting cells contributes to uterine immunity [47]. In the present study, intravaginal immunization was not effective in preventing uterine diseases, suggesting that mucosal immunization of the vagina, considering dose and composition used, does not affect the immunological status of the uterus. Nevertheless, it is important to highlight that the uterus is an immune tolerant environment during pregnancy [51], and this might have prevented the uterus to develop an immune response to the intravaginal vaccines. Further investigation is needed to evaluate if intravaginal vaccination administered prior to pregnancy would elicit a uterine immune response capable of prevent uterine diseases, and conclude if local synthesis of specific antibodies by the uterine mucosa is important for the prevention of puerperal metritis and endometritis in heifers.

In general, subcutaneous vaccination increased the serum levels of IgG against *E. coli*, FimH, *F. necrophorum*, LKT, *T. pyogenes*, and PLO. This suggests that there is a significant contribution of circulating specific IgG to the postpartum uterine immunity, a conclusion supported by previous studies. After intramuscular immunization with *Histophilus somni*, most of the IgG in uterine secretions of cattle at estrus were derived from serum [26]. Additionally, it has been reported that systemic immunization with *Campylobacter fetus* increased the IgG activity in the bovine reproductive tract. It is possible that IgG proteins work as opsonins in the bovine genital tract [27], hence contributing to phagocyte-dependent clearance of infection of the uterus.

It has already been documented that prevention of other diseases such as liver abscess and UTI are caused by some of the agents present as antigens within the vaccines tested in this study. It has been reported that a single injection of a bivalent *T. pyogenes* – *F. necrophorum* bacterin-toxoid reduced the prevalence of liver abscess when given to cattle entering a feedlot; reductions of 48.4% and 37.5% in the prevalence of liver abscess in the two trials reported [23]. It is known that *F. necrophorum* LKT is highly toxic to bovine PMNs [52], inducing apoptosis-mediated killing of them [53]; this toxicity is dose-dependent [54]. It is possible that immunizing the cows against LKT might have reduced the detrimental effect of this toxin on intrauterine PMNs, improving the ability of the innate immune system to eliminate bacterial infections from the uterus through phagocytosis. Recruited PMNs are key players in the immune defense of the uterus; reduced migration of PMNs 2 weeks before calving is associated with retained placenta [46], and lower phagocytic activity and oxidative burst capacity of PMNs are associated with occurrence of metritis and endometritis [43], [45].

Furthermore, it has been reported that systemic vaccination with FimH protects mice and cynomolgus monkeys from UTI [21], [55]. Mice that were immunized with FimH vaccines and challenged with an uropathogenic *E. coli* isolate exhibited a 100- to 1000-fold reduction in the number of organisms recovered from their bladders as compared to controls [21]. Additionally, cynomolgus monkeys immunized with FimH and further infected with a type 1-piliated *E. coli* isolate were protected against bladder infection, while control monkeys were affected with cystitis [55]. Although we did not observe a significant reduction in intrauterine presence of *E. coli* (the percentage of positive cows for *E. coli* culture was numerically lower for systemically vaccinated cows). Furthermore, based on our serological findings, systemic FimH immunization was an important factor for prevention of puerperal metritis.

This study evaluated the effect of multivalent vaccines; therefore, it is not possible to relate the effectiveness of the vaccines to any particular antigen. Published literature reported the multifactorial etiology of uterine diseases; therefore, we designed multivalent vaccines, aiming to successfully immunize cows against the most relevant known pathogens associated with uterine infections. Although our serological findings suggest that most of the antigens were partially important for the effectiveness of the subcutaneous vaccines, we do not know if certain antigens were potentially more important. Further research is needed to elucidate how important each antigen is for the effectiveness of the vaccines, and perhaps simplify the vaccine formulations.

In conclusion, the incidence of puerperal metritis was significantly decreased with prepartum subcutaneous vaccination with vaccines containing different combinations of proteins (FimH, LKT, PLO) and inactivated whole cells (*E. coli*, *F. necrophorum* and *T. pyogenes*). In contrast, intravaginal vaccination was not effective in decreasing the incidence of puerperal metritis. We can therefore suggest that commercial production of a vaccine against metritis may be feasible. Such a vaccine could become an integral part of a preventive strategy against metritis, leading to reduced incidence of the disease, reduced use of antibiotics and therefore alleviating both animal distress and the overall negative economic impact of metritis on the dairy industry.

## Supporting Information

Table S1 **Chemical composition (mineral and vitamins) of pre-fresh and lactating cows diets.** Pre-fresh diets were fed from 3 week prepartum through parturition and fresh diets were fed from parturition through week 35 postpartum.
(DOCX)
