# Supplementary material for: Subcutaneous Immunization with Inactivated Bacterial Components and Purified Protein of Escherichia coli, Fusobacterium necrophorum and Trueperella pyogenes Prevents Puerperal Metritis in Holstein Dairy Cows
Source: PLoS One. 2014 Mar 17;9(3):e91734. doi: 10.1371/journal.pone.0091734 (PMC3956715; doi:10.1371/journal.pone.0091734)
Supplement: Table S1 — Chemical composition (mineral and vitamins) of pre-fresh and lactating cows diets. Pre-fresh diets were fed from 3 week prepartum through parturition and fresh diets were fed from parturition through week 35 postpartum. (DOCX) [file pone.0091734.s001.docx]

| Chemical composition | Pre-fresh | | Fresh | |
| --- | --- | --- | --- | --- |
|  | As Fed | DM | As Fed | DM |
| Moisture % | 63.00 |  | 58.60 |  |
| Dry Matter % | 37.00 |  | 41.40 |  |
| Crude protein % | 5.10 | 13.60 | 6.50 | 15.70 |
| Soluble Protein % CP |  | 31.00 |  | 29.00 |
| Acid Detergent Fiber % | 11.20 | 30.20 | 8.50 | 20.50 |
| Neutral Detergent Fiber % | 17.00 | 45.80 | 13.80 | 33.00 |
| Calcium % | 0.49 | 1.32 | 0.36 | 0.86 |
| Phosphorus% | 0.15 | 0.39 | 0.18 | 0.43 |
| Magnesium% | 0.13 | 0.34 | 0.13 | 0.31 |
| Potassium% | 0.51 | 1.36 | 0.56 | 1.35 |
| Sodium% | 0.07 | 0.18 | 0.20 | 0.49 |
| Sulfur% | 0.16 | 0.42 | 0.11 | 0.27 |
| Copper ppm | 5.00 | 14.00 | 8.00 | 20.00 |
| Iron ppm | 113.00 | 304.00 | 78.00 | 188.0 |
| Manganese ppm | 32.00 | 85.00 | 26.00 | 63.00 |
| Selenium ppm | 0.22 | 0.60 | 0.33 | 0.80 |
| Zinc ppm | 25.00 | 66.00 | 42.00 | 103.0 |
| Molybdenum ppm | 0.40 | 1.10 | 0.50 | 1.20 |
